# Men’s Experiences and Psychological Outcomes of Nonsurgical Medical Penile Girth Augmentation: A Preliminary Prospective Study

**DOI:** 10.1093/asj/sjac243

**Published:** 2022-08-30

**Authors:** Gemma Sharp, Anne Nileshni Fernando, Jayson Oates, Peter McEvoy

**Affiliations:** senior research fellow and clinical psychologist Monash Alfred Psychiatry Research Centre, Monash University, Melbourne, Victoria, Australia interspecialty consulting editor for *Aesthetic Surgery Journal*; Ms Fernando is a research assistant, Monash Alfred Psychiatry Research Centre, Monash University, Melbourne, Victoria, Australia; Dr Oates is a facial plastic and cosmetic surgeon in private practice in Subiaco, Western Australia, Australia; Dr McEvoy is a professor of psychology and clinical psychologist, School of Population Health and enAble Institute at Curtin University, Bentley, Western Australia, Australia

## Abstract

**Background:**

The popularity of penile augmentation procedures is increasing, but investigation into men’s experiences with these procedures and their impact on psychological well-being is lacking.

**Objectives:**

The aim of this study was to investigate men’s experiences with nonsurgical medical penile girth augmentation and assess, based on valid psychological measures, the impacts these procedures have on psychological well-being.

**Methods:**

Men seeking to undergo a girth augmentation (n = 19) completed an online questionnaire prior to their procedure and 6 months later that contained standardized measures assessing impacts of the procedure, penile size self-discrepancy, body dysmorphic disorder, psychological distress, self-esteem, and body image–related quality of life. Girth size was also measured preprocedure and 6 months postprocedure for a subsample of men.

**Results:**

Almost half of the men reported positive impacts of “increased self-confidence” and “increased sexual pleasure” after their procedure. Despite an average girth increase of 3.29 cm, the men still perceived that their penile girth and length was less than what they should be or less than the ideal size after their augmentation procedure. However, this perceived discrepancy was significantly smaller than before their procedures. Prior to the procedure, the men who met diagnostic criteria for body dysmorphic disorder according to self-reported questionnaire (11%, n = 2/19) and clinical interview (7%, n = 1/15) lost this diagnosis at 6 months. There were no changes in psychological distress, self-esteem, or body image–related quality of life from pre- to postprocedure.

**Conclusions:**

Men report positive impacts on their lives after penile girth augmentation, but impacts on broader psychological well-being are mixed.

**Level of Evidence: 4:**

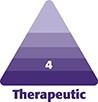

See the Commentary on this article here.

A sizable percentage of men are concerned about their penis size. A study of 25,592 men found that 45% of these men wanted a larger penis size, compared with only 38% of men who wanted to be taller in height.^1^ However, most studies suggest that men who have penile size concerns are actually in the normal population size range.^2^ Nevertheless, men are increasingly seeking medical solutions for what they perceive to be a less-than-ideal genital size.

Dissatisfaction with penis size has become a leading source of motivation for men to pursue penile augmentation procedures to ultimately increase the length and/or girth of their penis.^3^ While the rate of uptake of such procedures is rarely reported in the literature, anecdotally, there have been reports of an increasing number of procedures performed by medical professionals.^3^ Despite there being a wide variety of penile augmentation procedure methods, information is still emerging on the safety and efficacy of these.^4^ Furthermore, standardized procedures for penile augmentation are yet to be established.^3,5^ According to the literature, penile girth appears to be more important for sexual satisfaction than length, particularly from the perspective of sexual partners,^6^ and thus the focus of this study is penile girth augmentation.

Girth enhancement can be achieved through surgical and nonsurgical methods.^7–9^ Studies have found that nonsurgical methods such as the use of injectable materials generally result in lower complication and higher satisfaction rates compared with surgical methods.^8,10^ Whereas injectable materials such as paraffin, mineral oils, silicone, and autologous fat have seen the occurrence of multiple complications, studies reporting the use of injectable hyaluronic acid (HA) have shown fewer adverse effects and higher patient satisfaction levels.^3,5^ A recent systematic review investigating the efficacy and safety profiles of nonsurgical techniques also found that HA fillers were the safer technique associated with the greatest patient satisfaction, with patients declaring themselves “mostly satisfied.”^3^ In terms of girth increases, research has shown that HA fillers can yield enhancements of 1.7 to 3.92 cm, comparable to the outcomes for other types of currently available fillers.^10^

Although information on size increases and associated levels of patient satisfaction are available in the penile (girth) augmentation literature, these do not necessarily provide a comprehensive understanding of the patient’s experiences with the procedure and the impacts it had on their lives. It might be assumed that an increase in penis size would be accompanied by feelings of higher self-esteem, confidence, sexual satisfaction, and overall quality of life.^5,11,12^ Certainly, a desire to improve these areas is a commonly cited motivation men report when seeking penile augmentation.^13,14^ However, such factors are yet to be examined prospectively by means of standardized and validated measures. Poor-quality outcome measurement is common in this field of aesthetic clinical practice. Data are usually collected in a retrospective fashion, and are based on single-item, nonvalidated measures.^10,15^ These studies suggest that men may experience broader psychological benefits as a result of penile augmentation,^16^ but without prospective study designs based on validated measures, this is far from a certainty.

With respect to psychological functioning, a crucial area of investigation is body dysmorphic disorder (BDD) as this is the most common psychological disorder in individuals who seek cosmetic procedures.^17,18^ BDD is characterized by a preoccupation with a slight or perceived flaw in physical appearance that has a detrimental impact on social, occupational, and general life functioning.^19^ It is understandable that a person who believes they have a flaw in their appearance would seek cosmetic intervention for relief of their symptoms. However, rather than an alleviation of their psychological distress, individuals with BDD usually experience no change or a worsening of their symptoms after undergoing cosmetic treatment.^20^ Thus, BDD is generally considered to be a contraindication to cosmetic treatment.^18^ However, this is yet to be investigated for men seeking penile girth augmentation. Previous research in the field of penile augmentation has been limited by nonstandardized measures of BDD.^21^ Our own research has provided an estimate for the prevalence of BDD in men seeking penile girth augmentation at 11% to 14% (based on 2 validated BDD measures),^13^ which is at the higher end of the estimate across cosmetic procedure types of 5% to 15%.^18^ It is yet to be determined, by means of validated BDD measures, whether men experiencing BDD prior to their procedure maintain their diagnosis after undergoing penile girth augmentation.

In sum, the current preliminary study broadly aimed to examine the experiences of men undergoing nonsurgical HA-based penile girth augmentation using a prospective study design (preprocedure and 6 months postprocedure). In particular, the aim was to examine, by means of validated psychological measures, any changes in penile size self-discrepancy, BDD diagnosis, psychological distress, self-esteem, and body image–related quality of life from pre– to 6 months post–girth augmentation. Another study goal was to qualitatively examine men’s perceptions of the impacts of the penile girth augmentation on their lives.

## METHODS

### Participants

This prospective study recruited men from 3 private aesthetic surgery clinics in Australia who self-referred seeking nonsurgical HA-based penile girth augmentation as comprehensively outlined in Oates and Sharp.^5^ Inclusion criteria were: 18 years or older, no local infection, no permanent filler/implant, and realistic expectations based on clinical interview. Data for all men completing baseline measures were reported in Sharp et al.^13^ This study reports only on participants who underwent the penile girth augmentation and provided 6-month follow-up data. The data from 3 men were excluded from the study as they had not undergone a penile girth augmentation at the 6-month time point and this study specifically focused on the procedure outcomes.

### Demographic Variables

Questions were administered to assess demographic characteristics (age, marital status, sexual orientation, ethnicity, education, employment).

### Girth Measurements

Pretreatment flaccid penis girth measurements (midshaft) were obtained by the treating physician at initial consultation and also at 6 months postprocedure. A disposable paper tape measure at the mid-shaft position was utilized for these measurements.^22^

### Impacts of Penile Girth Augmentation

Participants were asked an open-ended (qualitative) question: “What impacts have the penile enhancement procedure had on your life? Please describe any positive or negative impacts.” Responses were read and coded for themes by the first 2 authors independently.^23^ The seven themes agreed on were: “increased self-confidence,” “increased sexual pleasure,” “general positive impact,” “aesthetic concerns,” “difficulties during sex,” “unexpected results,” and “no change.”

### Self-discrepancy Questionnaire

The Self-discrepancy Questionnaire (SDQ) includes a series of questions regarding a participant’s estimate of the size of their penis.^24^ Specifically, participants are asked for both length and girth estimates of their flaccid and erect penis in relation to other men for: (1) self-actual (what they believe their actual size is in relation to others); (2) self-ideal (how they would ideally like their penis to be in relation to others); and (3) self-should (what their penis should be in relation to others).

### Body Dysmorphic Disorder Questionnaire

The Body Dysmorphic Disorder Questionnaire (BDDQ) is a brief self-report screening measure for BDD based on DSM-5 (Diagnostic and Statistical Manual of Mental Disorders, 5th ed.) criteria.^19,25^ Questions assess appearance concerns and preoccupation, impacts of the preoccupation on the person’s life, and the duration of preoccupation each day. Item 2 also assesses whether the main appearance concern is that the person is not thin enough or that they might become too fat, in order to rule out an eating disorder rather than BDD (“eating disorder exclusion”). The BDDQ has demonstrated high sensitivity (100%) and a specificity of 92.3% on a cosmetic procedure–seeking sample.^26^

### MINI International Neuropsychiatric Interview 7.0.2 Body Dysmorphic Disorder Module

The MINI-BDD is a structured diagnostic interview that assesses the DSM-5 BDD criteria,^19,27^ including: (1) spending a lot time thinking about a defect or flaw in one’s appearance, (2) excessive worry, (3) recurrent thoughts comparing oneself to others or repetitive behaviours (eg, checking), and (4) whether these thoughts cause significant distress in important life domains. Positive responses to all of these questions are required for a BDD diagnosis.

### Kessler Psychological Distress Scale

The Kessler Psychological Distress Scale (K10) is a 10-item self-report measure of nonspecific psychological distress.^28^ The K10 has excellent internal consistency (Cronbach’s α = 0.93),^28^ it discriminates individuals with and without mental disorders,^29^ and has demonstrated reliability and validity across a range of populations.^30–32^ Symptoms are assessed over the previous 4 weeks on a Likert-type scale, from none of the time (1), a little of the time (2), some of the time (3), most of the time (4), and all of the time (5). A total score is calculated by adding ratings for all items, with a potential range of 10 to 50. The normative K10 mean [standard error] for men in the Australian National Survey of Mental Health and Wellbeing was 14.0 [0.1].^33^ Internal consistency was acceptable in this study (Cronbach’s α = 0.80 [preprocedure] and 0.76 [postprocedure]).

### Rosenberg Self-esteem Scale

The Rosenberg Self-esteem Scale (RSES) is a 10-item self-report measure of global self-esteem, with items rated on a 4-point scale from strongly disagree (1) to strongly agree (4).^34^ Half of the items are negatively worded and half are positively worded. The negatively worded items are reverse-scored so that higher scores reflect higher self-esteem. The scale has demonstrated good test-retest reliability (0.82-0.88) and adequate internal consistency (αs = 0.77-0.88).^35^ There is evidence that the RSES is unidimensional in nature and is typically represented by a total score with a potential range of 0 to 30.^36^ In a sample of 201 Australian men, there was a mean [standard deviation] score of 31.07 [5.15].^36^ Internal consistency in the current study was high (Cronbach’s α = 0.87 [preprocedure] and 0.94 [postprocedure]).

### Body Image Quality of Life Inventory

The Body Image Quality of Life Inventory (BIQLI) is a 19-item self-report scale measuring the impact of body image concerns on a broad range of life domains such as social functioning, sexuality, and emotional well-being.^37^ Items are rated on a 7-point scale from very positive impact on one’s life (+3), no impact (0), through to a very negative impact (−3). The BIQLI has demonstrated very high internal consistency (α = 0.95)^37^ and good test-retest reliability over a 2- to 3-week period (*r* = 0.79). There is evidence that the BIQLI is unidimensional,^38^ so can be represented by a total score. Internal consistency was very high in the current study both at preprocedure (α = 0.97) and postprocedure (α = 0.98). The BIQLI is negatively correlated with other measures of body image dissatisfaction and dysphoria, and positively associated with self-esteem.^39^ The BIQLI is calculated as a mean score across all items, with more positive scores reflecting a more positive body image.

### Procedure

Human Research Ethics Committee approval for this study was obtained from Curtin University, Perth, Australia (HRE2018-0268). From July 2018 to June 2021, patients who self-referred to 1 of 3 private aesthetic surgery clinics for penile girth augmentation completed an initial assessment with an aesthetic surgeon. Following the assessment, clinic reception staff provided patients who met the inclusion criteria with a brief information sheet and consent form to be contacted by the research team about a study designed to evaluate attitudes towards the penile girth augmentation procedure they were considering. Patients were informed that there was no commitment to participate if they signed this consent form, and that it was not necessary to proceed with the procedure. As stated above, a minority group (n = 3) who completed the 6-month follow-up questionnaires but did not proceed with the augmentation procedure were excluded from the present study, which focused on procedure outcomes. Patients who completed the consent form were contacted by research staff independent of the clinic to provide more detailed information in verbal and written formats, and another consent form to participate in the study itself.

Consenting patients were assigned a unique study identification number and were sent a weblink to an online survey containing the baseline validated measures and then another weblink approximately 6 months postprocedure. A blank copy of the survey is available in Sharp et al.^13^ The survey was completed anonymously and included demographic details and motivations for considering penile augmentation, and the SDQ, BDDQ, K10, RSES, and BIQLI. As reported in Sharp et al,^13^ there was an error in the administration of the Cosmetic Procedure Screening Scale for Penile Dysmorphic Disorder (COPS-P)^21^ at the preprocedure time point (not all items administered) which was repeated at the 6-month follow-up and so these incomplete data were excluded from the present study. Once the online measures were completed, the study research assistant arranged a time to contact the participant to complete the BDD module of the MINI by telephone. All participants were contacted again 6 months after they completed the baseline measures to complete a second and final online survey, which included the open-ended question on the impacts of the procedure, and the SDQ, BDDQ, K10, RSES, BDDQ, and BIQLI. Participants were again contacted to complete the MINI-BDD by telephone. A list of support services was provided in case participants were experiencing distress, and they received a AUD$50 (∼US$35) Amazon voucher to compensate them for their time.

### Analytical Procedure

The data were analyzed with SPSS version 27.0 (IBM SPSS, Inc., Chicago, IL). Sample characteristics were reported descriptively. For the 6-month postprocedure time point, paired *t* tests were used to examine differences in perception of actual penis size (erect length, nonerect length, girth) and what an individual believed their penis size should be, and their ideal size (as measured by the SDQ). Mean difference scores for actual size vs “should be” and actual vs “ideal” size were also calculated for pre- and postprocedure time points and these differences in mean difference scores across time examined via paired *t* tests. Finally, paired *t* tests were also used to examine differences from pre- to 6 months postprocedure on measures of distress (K10), self-esteem (RSES), and body image quality of life (BIQLI) as well as penile girth measurement. Cohen’s *d* statistics were calculated to examine the magnitude of these differences.

## RESULTS

### Preprocedure Sample Demographics

The sample consisted of 19 male pre–penile girth augmentation patients. As seen in Table 1, the age of the sample ranged between 27 and 68 years (mean, 44.58 [11.02] years). Most of the participants were married and identified as heterosexual. Additionally, most of the men were of Australian ancestry and were engaged in full-time work.

**Table 1.** Pre–Girth Augmentation Procedure Demographic Characteristics (*n* = 19)

Characteristic	n (%)
Age (years)	44.58 [11.02] (27-68)
Marital status	
ȃSingle	4 (21)
ȃMarried	9 (47)
ȃDivorced	2 (11)
ȃWidowed	1 (5)
ȃSeparated	3 (16)
Sexual orientation	
ȃHeterosexual	16 (84)
ȃHomosexual	2 (11)
ȃBisexual	1 (5)
Ethnicity^a^	
ȃAustralian	14 (74)
ȃUK (English, Irish, Scottish)	4 (21)
ȃSouthern European (Italian, Greek, French)	4 (21)
ȃAsia (Chinese, Indian, Indonesian)	2 (11)
ȃBrazilian	1 (5)
Highest level of education	
ȃHigh school (up to year 10)	3 (16)
ȃHigh school (up to year 12)	3 (16)
ȃTAFE (or similar)	2 (11)
ȃApprenticeship	2 (11)
ȃUniversity (bachelor’s degree)	6 (32)
ȃUniversity postgraduate masters (coursework or research)	2 (11)
ȃUniversity doctoral degree	1 (5)
Work status	
ȃNot working at the moment	1 (5)
ȃPart time work (15-34 hours a week)	1 (5)
ȃFull-time work	17 (90)
Psychological distress (K10)^b^	
ȃLow	13 (68)
ȃModerate	6 (32)
BDD from BDDQ	
ȃNo	14 (74)
ȃYes (without eating disorder exclusion)	3 (16)
ȃYes (with eating disorder exclusion)	2 (11)
BDD from BDD-MINI (*n* = 15)	
ȃNo	14 (93)
ȃYes	1 (7)

Values are mean [standard deviation] (range) or n (%). BDD, body dysmorphic disorder; BDDQ, Body Dysmorphic Disorder Questionnaire; K10, Kessler Psychological Distress Scale; MINI, Mini International Neuropsychiatric Interview; TAFE, technical and further education. ^a^Multiple options could be selected and therefore total number >19. ^b^K10 categories are based on reported categories in Slade et al: 10-15, low; 16-21, moderate.^33^

### Changes in Girth Measurements and Complications

Seven participants (37%) completed both pre- and postprocedure penile girth size measurements. The mean girth (flaccid) preprocedure was 9.50 [1.08] cm and this increased to 12.79 [1.90] cm 6 months postprocedure. This mean increase of 3.29 cm was statistically significant (*t* = −7.50, *P* < 0.001, Cohen’s *d* = 2.13). None of the 19 men who underwent the procedure experienced a complication.^40^

### Perception of Impacts of Penile Augmentation

The various impacts on the lives of the men who underwent penile girth augmentation are shown in Table 2. Importantly, as this question was asked in an open-ended response format, most participants nominated several different impacts on their lives, with some nominating both positive and negative impacts within the same response. Specifically, almost half of the participants (n = 9, 47%) reported a positive impact on their self-confidence and/or an increase in sexual pleasure for themselves or partner(s) (n = 8, 42%). Some reported a more general positive impact on their lives without specifying further (n = 4, 21%). “Aesthetic issues” was the most common negative impact reported (n = 4, 21%), which appeared to focus on the distribution of the HA filler. “Difficulties during sex” and “unexpected results” were the next most common negative impacts. A minority of participants reported “no change” to their lives after penile girth augmentation (n = 2, 11%).

**Table 2.** Impacts of Penile Girth Augmentation by Theme (*n* = 19)

Theme	Example	n (%)	n (%) as sole reason
Increased self-confidence	“A marvellous improvement to my confidence …” “It has given me a new-found confidence in and out of the bedroom”	9 (47)	3 (16)
Increased sexual pleasure	“… it has heightened my sexual pleasure both in how my penis feels and my brain stimulation” “It’s been great some extra pleasure for my sexual partner”	8 (42)	2 (11)
General positive impact	“Very positive impact on my life” “Generally positive”	4 (21)	0 (0)
Aesthetic concerns	“Volume was definitely noticeable but the filler never spreads evenly or to the desired shape” “… shaft of the penis looks puffy …”	4 (21)	1 (5)
Difficulties during sex	“Found it harder to ejaculate …”	1 (5)	0 (0)
Unexpected results	“Not the result I expected penis is lumpy and withdraws further up into the sheath”	1 (5)	0 (0)
No change	“Not really any impact”	2 (11)	2 (11)

Percentages do not sum to 100% because participants reported impacts that were coded into multiple themes.

### Self-discrepancy for Penis Size

Post-girth augmentation perceived “actual,” “should be,” and “ideal” penile sizes (erectile length/nonerectile length/girth) are shown in Table 3. On average men perceived their “actual” size to be above average (>50th percentile) at postprocedure for all 3 size dimensions, but their “should be” and “ideal” sizes were even larger. The differences between perceived “actual” and “should”/“ideal” for each size dimension were statistically significant with moderate to large effect sizes.

**Table 3.** Post-Penile Girth Augmentation Self-discrepancy Questionnaire Score (as Percentiles) and *t* Test Scores of Differences (n = 18)

Size dimension	Mean [SD]	Minimum	Maximum	Actual vs	*t*	*P*	Cohen’s *d*
Erectile length							
Actual	57.28 [18.02]	31	89	—			
Should	73.33 [8.64]	57	88	−16.06	−5.01	<0.001	1.14
Ideal	77.00 [12.39]	58	100	−19.72	−4.91	<0.001	1.28
Nonerectile length							
Actual	52.50 [17.67]	28	82	–			
Should	66.06 [14.45]	35	97	−13.56	−4.51	< 0.001	0.84
Ideal	71.17 [17.05]	35	100	−18.67	−4.55	< 0.001	1.08
Girth							
Actual	59.78 [22.52]	18	97	—			
Should	70.17 [14.54]	36	97	−10.39	−3.27	0.005	0.55
Ideal	74.33 [17.34]	39	100	−14.56	−4.07	< 0.001	0.72

SD, standard deviation.

The size discrepancies between “actual” and “should,” and “actual” and “ideal” were also compared at the pre- and postprocedure time points (see Table 4). The discrepancies were significantly smaller at postprocedure compared with preprocedure for girth and flaccid length, but not erect length.

**Table 4.** Comparisons in Self-discrepancy Questionnaire Scores at Pre- and 6 Months Post-Penile Girth Augmentation and *t* Test Scores (*n* = 18)

	Pre-penile girth augmentation (mean [SD])	Post-penile girth augmentation (mean [SD])	*t*	*P*	Cohen’s d
Erectile length					
Actual vs should	20.33 (11.31)	16.06 (13.61)	1.37	0.19	0.34
Actual vs ideal	20.78 (8.99)	19.72 (17.05)	0.29	0.77	0.08
Nonerectile length					
Actual vs should	23.83 (13.50)	13.56 (12.77)	2.85	0.01	0.78
Actual vs ideal	29.83 (18.02)	18.67 (17.41)	3.33	0.004	0.72
Girth					
Actual vs should	22.44 (15.91)	10.39 (13.49)	2.53	0.02	0.82
Actual vs ideal	27.78 (16.11)	14.56 (15.17)	3.28	0.004	0.85

SD, standard deviation.

### Body Dysmorphia Disease

As seen in Table 1, 3 patients met criteria for BDD preprocedure based on the BDDQ when the eating disorder exclusion was not applied (“Is your main concern with your appearance that you aren’t thin enough or that you might become too fat?”; 16%), with 2 patients (11%) meeting criteria when this criterion was applied. At 6 months postprocedure, 2 patients met criteria when the eating disorder exclusion was not applied (11%), but this became zero patients when this criterion was included. For the MINI-BDD module (see Table 1), 1 patient out of the 15 (7%) who could be contacted by telephone to complete the interview met criteria preprocedure, but at follow-up this patient no longer met criteria.

### Psychological Distress, Self Esteem, and Body Image Quality of Life

As seen in Table 5, based on the K10, RSES, and BIQLI, there was a decrease in mean psychological distress and increases in mean self-esteem and body image-related quality of life from preprocedure to 6 months postprocedure. However, none of these changes reached statistical significance and all had small effect sizes.

**Table 5.** Psychological Distress, Self-esteem, and Body Image Quality of Life Scores at Pre- and 6 Months Post-Penile Girth Augmentation, and *t* Test Scores

Variable	Pre-penile girth augmentation (mean [SD])	Range	Post-penile girth augmentation (mean [SD])	Range	*t*	*P*, Cohen’s d
K10, n = 19	14.74 (3.89)	10-25	14.32 (3.70)	10-21	0.47	*P* = 0.64, *d* = 0.11
RSES, n = 19	22.11 (5.45)	10-30	23.79 (6.00)	13-30	−1.93	*P* = 0.07, *d* = 0.29
BIQLI, n = 18	0.61 (1.55)	−1.53 to 2.63	1.19 (1.34)	−1.53 to 2.74	−1.32	*P* = 0.21, *d* = 0.40

SD, standard deviation, K10, Kessler 10-Item Distress Scale; RSES, Rosenberg Self-esteem Scale; BIQLI, Body Image Quality of Life Inventory.

## DISCUSSION

This preliminary study is the first to prospectively and comprehensively examine, by means of validated psychometric measures, the experiences and psychological outcomes of men who have undergone a nonsurgical HA-based penile girth augmentation. As such, the study has started to provide crucial insights into men’s experiences with this increasingly popular field of aesthetic practice. Specifically, around half of the men reported positive impacts on their self-confidence and/or sexual pleasure when provided with an open-ended response format, but a sizable percentage reported less positive impacts, particularly concerns with the aesthetic outcomes. The men also still perceived their postprocedure penile girth and length to be smaller than their ideal size, but this discrepancy had reduced significantly as a result of the girth augmentation. None of the men still met diagnostic criteria for BDD after the procedure irrespective of the diagnostic measure employed. Finally, there were no significant changes in broader psychological distress, self-esteem, and body image-related quality of life from pre- to 6 months post–girth augmentation.

Although the present study focused on psychological outcomes, change in penile girth was measured by the treating physician for a subsample of the men. Before the procedure, average flaccid girth was within the normal size range (8.5-10.5 cm),^9^ but this increased to, on average, well above the normal size range (12.79 cm) approximately 6 months after the procedure. The average 3.29-cm increase is at the upper end of size increases reported in previous systematic reviews for HA-based girth augmentations.^3,10^ It should be noted that fewer than half of the men returned for a follow-up consultation (although all were invited) and so the change in girth size could not be determined for all men involved in the present study. The authors could have potentially asked the participants themselves to measure their girth as part of the postprocedure questionnaire, but for the sake of accuracy, clinician measurement was deemed optimal. Although the increase in girth size was on the higher end for the men in the present study, previous research suggests that for this increased girth size to be maintained, further HA filler is required around 18 months later, so this is not a permanent outcome.^5^

It is interesting to note that although the men in our sample seemingly possessed a penis with a girth well above the normal size range after augmentation, they still reported that they were smaller than what they should be or ideally would be. This was also the case for their “should be” and “ideal” erect and nonerect length sizes. The discrepancy for length is possibly to be expected given that the procedure specifically aims to increase girth. However, it does suggest that the men undergoing these procedures may be underestimating their own penile sizes and overestimating those of other men.^2^ Such a phenomenon is supported by previous research showing that men’s penis size attitudes are influenced by media sources such as pornography where the male actors are chosen on the basis of their very large penises.^41,42^ Importantly, perceived size discrepancies were significantly smaller for girth and nonerect length (but not erect length) at postprocedure compared with preprocedure. Thus, the men in the present study were at least closer to their aspirational sizes as a result of their girth augmentations. This is possibly a reason for previous research reporting moderately high levels of patient satisfaction after girth augmentation, but not complete satisfaction.^3,8,16^ It will be important for aesthetic medical practitioners to discuss with patients at initial consultations the size increases they can expect after augmentation. This could potentially be supplemented with the use of 3-dimensional models to assist patients with visualizing the potential outcome.^43^

Nevertheless, the present study aimed to substantially extend upon previous research which has generally used simple and unvalidated satisfaction measures in predominantly retrospective study designs. In this study, men were asked to state how their lives had been impacted by girth augmentation and to prospectively answer a series of validated psychometric measures. This provided much-needed nuance to the interpretation of the outcomes. For example, around half of the men reported that their self-confidence and/or their sexual pleasure was increased. As reported in our previous research, 47% of men were motivated to undergo the procedure to improve self-confidence and 33% to improve their sexual function/pleasure.^13^ Thus, it appears that at least some of the men experienced the outcomes they desired. However, a smaller but sizable percentage of men also offered impacts on their lives that were more negative. These predominantly focused on aesthetic concerns about the HA filler not spreading evenly throughout the penis. As discussed above, the men in the study possibly wanted even more HA filler injected to increase their girth size further, but aesthetic outcomes are still very important to patients. Clearly, care must be taken by the treating physician to optimize the amount of filler injected vs the appearance of the penis.^5^ Other concerns included difficulty with ejaculation (which would unlikely be rectified through girth augmentation)^14^ or no changes to the men’s lives at all. We encourage aesthetic practitioners to collect feedback from patients in open-ended response formats (not only close-ended, satisfaction-based questionnaires) because such feedback is crucial for optimizing surgical techniques and the patient experience.

The present study also provided useful information for BDD prevalence in men pre- and post-penile girth augmentation based on a validated self-report measure in the BDDQ and a clinician-administered measure in the MINI-BDD module. The 2 men (11%, n = 2/19) who met diagnostic criteria according to the BDDQ and 1 man (7%, n = 1/15) according to the MINI-BDD module preprocedure lost their diagnosis at the 6-month follow-up time point. With a small sample, these results must be interpreted with great caution, but potentially BDD may not necessarily be a contraindication for nonsurgical medical penile girth augmentation. This may be because the outcomes of a penile girth augmentation (ie, increased girth size) are less ambiguous than for other procedures such as rhinoplasty (ie, change shape/size of the nose). Such a “loss of BDD diagnosis” has been previously reported for female aesthetic genital surgery patients similarly assessed with validated and standardized measures of BDD.^44,45^ However, it is possible in the longer term that patients who have “lost” their diagnosis may become obsessively concerned with another body part and meet BDD diagnostic criteria again.^44^ Future prospective long-term research is definitely needed and would benefit from the use of a more in-depth structured clinical interview for BDD (eg, the Structured Clinical Interview for DSM Disorders)^46^ rather than the briefer BDDQ and the MINI-BDD module. We encourage aesthetic practitioners to additionally employ their in-depth clinical interview skills to examine potential BDD symptoms at the initial consultation and after aesthetic intervention.

In addition to BDD, the impacts of girth augmentation on psychological distress, self-esteem, and body image–related quality of life were also examined by means of standardized and validated measures. There were no significant changes in any of these psychological well-being measures. Notably, the men were not highly psychologically distressed preprocedure, according to the K10 scale, and so there was not much room for improvement in this capacity. However, as discussed above, the impacts of the procedure on the men’s lives (when asked in an open-ended format) were a mixture of positive and negative, even within the same patient. Thus, although some men did report improvements in the measures of psychological distress, self-esteem, and body image–related quality of life, this was not consistent across the sample. It may also be unrealistic to expect major changes from a simple change in penis size in multidimensional psychological constructs, such as self-esteem, which are influenced by a number of factors. Such findings are consistent with other forms of aesthetic surgery whereby the patient’s distress surrounding the operated body part is alleviated, but they tend not to experience significant changes in other aspects of their lives.^47–53^ Nevertheless, this study provides an important foundation for pre-/postprocedure scores for commonly used measures, ie, the K10, RSES, and BIQLI, in a group of penile augmentation patients.

Some limitations should be taken into account when interpreting the results of this study. As a preliminary report, the sample size was small. As such, there was not the statistical power to detect smaller effect sizes or conduct statistically sound subgroup analyses (eg, based on BDD diagnosis). Moreover, only just over a third of men returned for a consultation with their treating physician where any changes in their penis girth size could be measured, so the association between the magnitude of size increase and psychological outcomes could not be investigated. The researchers did conduct the study over several years in the hope of recruiting a larger sample size: the small number of participants is indicative of the challenge of involving men in research who are seeking and undergoing penile girth augmentation.^42^ Furthermore, the men included in this study may not have been representative of the population of men seeking penile augmentation more broadly. The men in our sample were seeking a specific HA-based injectable girth augmentation in clinics based only in Australia. There was also no control group in which any changes in measures over time could be examined and compared. This should be addressed in future research.

## CONCLUSIONS

Notwithstanding its limitations, this study was able to provide novel insights and serves as an important platform for future research into the experiences and psychological outcomes of men who undergo nonsurgical HA-based penile girth augmentation. Along with an average increase in girth size of 3.29 cm, around half the men reported positive impacts on their lives, particularly self-confidence and sexual pleasure. However, a sizable percentage reported some less positive results, especially concerns with the aesthetic outcomes. The men also still perceived their girth and length to be smaller than their ideal size but a significant improvement on their preprocedure size perceptions. None of the men still met diagnostic criteria for BDD after the procedure irrespective of the diagnostic measure employed. Finally, there were no changes in broader psychological distress, self-esteem, and body image–related quality of life after undergoing girth augmentation. Due to the preliminary nature of the study and the small sample size, the findings of the study should be interpreted with a degree of caution. However, the study results will potentially assist clinicians in their psychological care of men seeking a penile augmentation and help to inform their clinical decision-making.
